# An Automated Hydrodynamically Mediated Technique for Preparation of Calibration Solutions via Capillary Electrophoresis System as a Promising Alternative to Manual Pipetting

**DOI:** 10.3390/molecules26206268

**Published:** 2021-10-16

**Authors:** Małgorzata Gołąb, Michał Woźniakiewicz, Paweł M. Nowak, Paweł Kościelniak

**Affiliations:** Laboratory for Forensic Chemistry, Department of Analytical Chemistry, Faculty of Chemistry, Jagiellonian University, Gronostajowa 2, 30-387 Krakow, Poland; mgolab@doctoral.uj.edu.pl (M.G.); pm.nowak@uj.edu.pl (P.M.N.); pawel.koscielniak@uj.edu.pl (P.K.)

**Keywords:** automation, calibration, capillary electrophoresis, calibration solutions

## Abstract

In this paper, a novel procedure for preparing calibration solutions for capillary electrophoresis (CE)-based quantitative analysis is proposed. Our approach, named the automated hydrodynamically mediated technique (AHMT), uses a capillary and a pressure system to deliver the expected amount of working solution and diluent directly to a sample vial. As a result, calibration solutions are prepared automatically inside the CE instrument, without any or with minimal manual operation. Two different modes were tested: forward and reverse, differing in the direction of hydrodynamic flow. The calibration curves obtained for a model mixture of analytes using AHMT were thorough compared to the standard procedure based on manual pipetting. The results were consistent, though the volume of obtained calibration solutions and the potential risk of random errors were significantly minimized by AHMT. Its effectiveness was further enhanced by the application of SCIEX^®^ nanoVials, reducing the actual volume of calibration solutions down to 10 μL.

## 1. Introduction

In recent years, there has been increasing interest in methods focusing on the automation of sample pre-treatment and analysis, as well as implementation of the rules of ‘green chemistry’ [1]. Automation enables lessening reagents’ consumption, hence decreasing the amount of chemical waste. Usually, it also shortens the time of the whole analysis process and limits the influence of the operator on basic validation parameters, such as accuracy, precision, repeatability, and linearity. Furthermore, since an automated system is more isolated from the environment, sample contamination or alteration is minimized and analyst safety is highly improved while working with hazardous substances [2,3]. For the purpose of automation sample pre-treatment and analysis, flow-based methods are mostly exploited [4,5,6]. The vast majority of papers related to automation of separation techniques focus on sample pre-treatment [7,8], rather than online preparation of calibration solutions. Nevertheless, there are some commercially available online dilution systems dedicated to ICP-MS [9], ICP-OES [10], and HPLC [11,12]. Such systems enable preparation of solutions at desired concentration levels by setting the dilution factor using the appropriate software provided to program the dilution procedure, as well as later peaks integration and the automatic calibration procedure. To the authors’ knowledge, in capillary electrophoresis (CE), there has been only one reference describing a direct, fully automated calibration procedure using a commercially available CE instrument that is coupled with a continuous flow system (CFS) via a programmable arm. Arce et al. [13] proposed a device for preparation of calibration solutions composed of peristaltic pumps, a mixing coil, and a programmable arm, which directly transfers obtained standard sample vials to the inlet table of the CE instrument.

In this work, we show for the first time how to prepare calibration solutions using only a commercially available CE instrument and unmodified software. The original procedure for automatically transferring expected volumes of solutions, named automated hydrodynamically mediated technique (AHMT), was developed and validated. The application of the presented approach may cover either forensic or field analyses, as well as determination of inorganic cations or anions. Furthermore, it can provide an opportunity to introduce analysis kits equipped with all necessary solutions and a description of the calibration procedure.

## 2. Results and Discussion

### 2.1. Development of the AHMT Procedure

The developed AHMT procedure is based on the use of the pressure system of the CE apparatus to transfer the expected volume of the solution via capillary. In this approach, two parameters must be adjusted: applied pressure and duration time. The initial experiment aimed to prepare a 50 µg/mL calibration solution, namely to obtain a 2-fold diluted working solution spiked with IS (WS). For this purpose, WS and a 10-times, water-diluted background electrolyte spiked with IS (DBGE) were transferred to the empty PCR vial for 10 min with the pressure of 137.90 kPa (20 psi) each. Further experiments showed that the 10 min transport time of the solution via capillary proved to be the minimal time to obtain a good repeatability of calibration solution concentration values (CV < 3%, data not shown). The construction (calculation of time and pressure values) of remaining calibration solutions relied on maintaining constant total volume (sum of volumes for WS and DBGE) with the assumption of equal viscosities of WS and DBGE. In practice, it meant that the sum of multiplications of time and pressure for WS and DBGE was constant for all prepared solutions. As long as the transfer time of WS for all calibration solutions was the same (10 min), only the pressure value had to be changed proportionally to give the required concentration. The volume of DBGE was adjusted on the same principle. Using the pressure within the range of 17.24 kPa (2.5 psi)–206.84 kPa (30 psi) and appropriate manipulation with the pumping time to keep the operation in reasonable time (up to 15 min), calibration solutions were prepared. The parameters for construction of calibration solutions are collected in Table 1.

The above-mentioned conditions enabled the experiment to yield about 100 µL of each calibration solution. In the next step, the possibility of the application of nanoVials (SCIEX^®^) for the automated preparation of calibration solutions was evaluated. The nanoVials are designed to facilitate repeatable injections of samples of volume down to 5 µL. Therefore, calibration solutions were prepared in nanoVials with some necessary amendments: WS was added as the second solution to minimize possible errors caused by uneven mixing, and time-pressure conditions had to be changed. Conditions are collected in Table 1, and using the pressure within the range of 3.45 kPa (0.5 psi)–26.20 kPa (3.8 psi) allowed us to decrease the volume of calibration solutions down to 10 µL.

**Figure 1:**
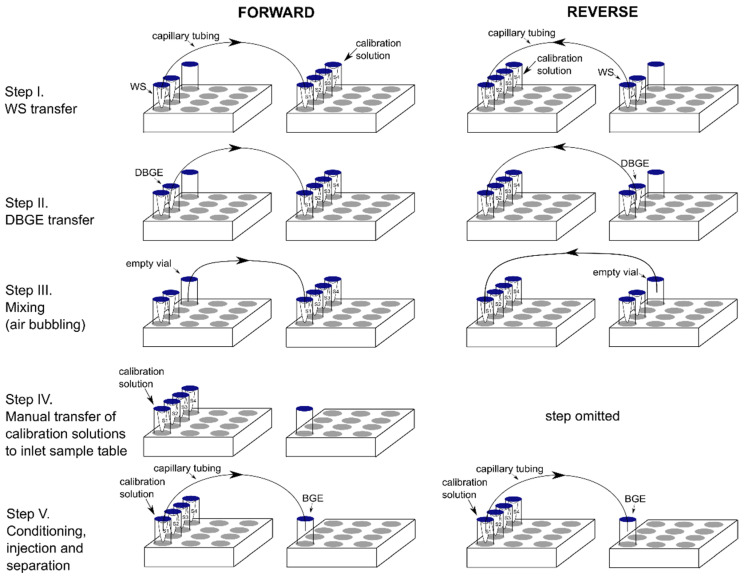
Scheme comparing performance of two modes of AHMT.

AHMT was performed in two modes: ‘reverse’ (R) and ‘forward’ (F), differing in the direction of flow inside the capillary (see Figure 1). In both modes, one sample tray of the CE instrument was used as the course of reagents, while the other one served as the collector. In R mode, PCR vials, placed in sealed buffer vessels were filled with WS or DBGE (200 µL each) and situated in the outlet sample tray, while the empty PCR vials were similarly assembled and put in the inlet sample tray, ready for calibration solution collection. The F mode required a reversion of vial position in the sample trays (step IV, Figure 1).

To avoid carryover of one of the solutions, a rinsing procedure of the capillary was proposed. Initially, the capillary was rinsed (137.90 kPa, 20 psi) with water (1.5 min) and methanol (1 min), then dried with air flow (1.5 min) and filled with WS for 2 min. Next, the tip of the capillary was cleaned by immersion in a vial filled with water. Afterwards, the appropriate volume of WS was added to all the empty PCR vials placed in the sample tray, where the calibration solutions were constituted (step I, Figure 1). In the next step, the capillary was rinsed with water (3 min) and DBGE (2 min) to make a capillary filled with the second solution. Before putting the capillary to the next vial, the tip was cleaned, as described above. Once the addition of DBGE to all vials was completed (step II, Figure 1), the capillary was rinsed with water and methanol and dried with air for 1.5 min each. To avoid a concentration gradient in the collecting vials, the vial content was gently mixed by air bubbling as a result of applying pressure of 206.86 kPa (30 psi) for 1 min to the empty vial, while the other end of the capillary was immersed in a PCR vial filled with constituted calibration solution (step III, Figure 1). Finally, the separation step proceeded (see Section 3.4).

### 2.2. Comparative Studies

Calibration solutions (6.25, 12.5, 25.0, 37.5, 50.0, 75.0 µg/mL; non-diluted WS was also used for calibration purposes) were prepared according to two methodologies: AHMT, using the capillary electrophoresis instrument as the pipetting robot, and external, by manual pipetting, particularly for the purpose of verification of the automatic approach. After the preparation of calibration solutions, they were analyzed with the CE method (see Section 3.4).

Since the variation in peak area is one of the major problems encountered in CE analysis, the repeatability of the analytical signal may be significantly improved using time-corrected peak area (TCPA) values [14]; this methodology was also implemented in this study. Additionally, these results were corrected by the means of internal standard; thus the analytical signal was described as the ratio TCPA_Analite_/TCPA_IS_. For each calibration solution, CE analysis was performed in triplicate. Then the analytical signal vs. concentration graphs were plotted, and the linear regression functions were calculated. With the analysis of standard residuals enabled, outliers were discarded whenever they did not meet the conditions [15]. Next, for each calibration solution, the average value of the time-corrected peak areas (taking into account only the remaining repetition points) was calculated, and the weighted regression was performed ($w=\frac{1}{x}$). The evaluation of the consistency between manual and automatic approaches required an experiment to be performed within the same working day. It is highly beneficial for the stability of the system, but it involves some limitations due to prolonged time of analysis. To fit in the single working day, only a single execution of each approach was performed. The same methodology was applied on the other day using another CE instrument of a previous generation (P/ACE MDQ, Beckman Coulter) to confirm the efficacy of the developed procedure. The results are collected in Table 2.

Evaluation of obtained results revealed that the calibration plots prepared in the automatic process inside the CE instrument provided concentration values that did not significantly differ from theoretical concentration values, with a few exceptions for the lowest concentration level (see Table S1 in Supplementary Materials). The relative error did not exceed 4%, except at the lowest calibration point, where it did not exceed 13% in reverse (R) mode. Such an observation strongly supports the scientific hypothesis that automatic preparation of calibration solutions using the capillary and the CE pressure system is reliable.

To further assess the compliance of the models obtained with the use of automatic and manual approaches, the plot of the concentrations determined for the automatic mode with respect to the concentration obtained using the manual mode was prepared and the Deming regression was applied.

Deming regression is a type of linear regression that takes into account errors of both variables. It is commonly used in comparative studies, where compared variables are the subject of random error, which is not taken into account in the least-squares regression method [16,17]. Examples of use of this type of regression might be found in clinical chemistry [18,19] and bioanalytical analysis [20]. Application of Deming regression requires a calculation of the ratio between squared standard deviations of both variables [21]. For this purpose, the mean standard deviations for all results obtained with the given mode were also determined and used as the standard error. The obtained parameters of fitted curves with uncertainties are summarized in Table 3, showing an excellent compliance between automatic and manual standards preparation. This experiment proves that the pressure and flow system of the CE instrument is efficient enough to be used as the pipetting robot for preparation of calibration standards.

**Figure 2:**
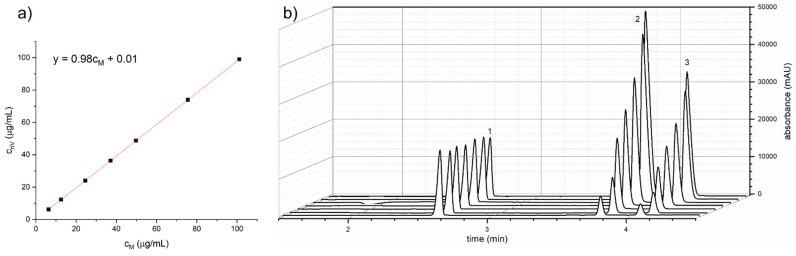
(**a**) Results of Deming regression for comparison concentrations obtained from the nanoVials in reverse mode (c_nV_) and the manual (c_M_) approach; (**b**) Electropherograms obtained by R mode in nanoVials (IS calibration method). 1: procaine (IS), 2: prilocaine, 3: bupivacaine. BGE: 103.9 mmol/L of H_3_PO_4_ and 37.66 mmol/L of TRIS; CE conditions: capillary length 30.5 cm (20.5 cm to the detector), i.d. 50 μm; applied voltage: +15 kV; hydrodynamic injection 0.5 psi for 6 s.

Since limitation of sample consumption is of particular importance in bioanalysis, growing interest in the application of nanoVials may occur. The potential benefit of nanoVials’ use for the preparation of automatic calibration solutions should be also taken into consideration, particularly whenever expensive analytical standards are required; thus, it is of high demand to lower reagents’ consumption down to a microliter level. In Beckman Coulter CE systems (currently offered by SCIEX^®^), the injection from a sample of 10 µL volume is not possible in regular PCR vials as the capillary end is above the liquid level (see Figure S1 in Supplementary Materials to compare the 10 µL sample in PCR and nanoVials). To confirm the possibility of the use the nanoVials in AHMT ‘reverse’ mode, procedures using nanoVials (nV), PCR vials (R), and additionally performed manual calibration (M) were evaluated. Determination coefficients calculated for every calibration plot obtained using AHMT in nanoVials were satisfactory, R^2^ > 0.9995 (see Table 2). The compliance between the manual, AHMT reverse mode in PCR vials and nanoVials was evaluated using the above-described linear models. The results of the Deming regression and concentrations’ relative errors are presented in Figure 2a, Table 3, and Table S2, respectively. 

Application of the nanoVials restrained the necessary amount of working standard solution down to 200 µL for preparation of seven calibration solutions (non-diluted WS was also used for calibration purposes). This saving is significant, since in the AHMT using PCR vials and the manually prepared solutions, a greater than double amount of standard solution is consumed. The latter is also affected by the risk of increased random errors due to pipetting of tiny volumes. Such a reduction in analytical standard consumption is of great importance in terms of cost effectiveness and green chemistry. It is worth emphasizing that, in the regular CE method (injection at the anodic capillary end and the separation with the positive polarity of the electrodes), the ‘reverse’ mode of AHMT should be recognized as more applicable than the ‘forward’ mode because once the sequence is initiated, no further action is required until the review and processing of acquired electropherograms. Electropherograms obtained by R mode using nanoVials are presented in Figure 2b.

## 3. Materials and Methods

### 3.1. Chemicals and Reagents

Procaine, prilocaine, bupivacaine, methanol (HPLC grade), and 2-amino-2-(hydroxymethyl)propane-1,3-diol (TRIS) were purchased from Sigma-Aldrich (St. Louis, MO, USA). Sodium hydroxide 30% solution was supplied by Avantor Performance Materials Poland S.A. (Gliwice, Poland). Phosphoric acid 85% solution was provided by Merck (Darmstadt, Germany). Ultrapure water (18.2 MΩ·cm, 3 ppb TOC) was generated in our laboratory in a Milli-Q system by Merck-Millipore (Darmstadt, Germany).

### 3.2. Preparation of Stock and Standard Solutions

Stock solutions of procaine, prilocaine, and bupivacaine (20 mg/mL), the solution of procaine (2 mg/mL), and a mixture of all above-mentioned substances (2 mg/mL each) were prepared in methanol. Stock solutions were stored in amber glass vials in a freezer. Other solutions were kept at +4 °C.

Every working day, a fresh working standard solution (WS, 100 µg/mL) was prepared using a 2 mg/mL mixture of analytes and an appropriate volume of 10 times water-diluted background electrolyte (DBGE). Initial experiments revealed (data not shown) that performing an internal standard calibration is recommended which allows for the determination of two analytes: prilocaine and bupivacaine in this experiment. For this purpose, DBGE was additionally spiked with procaine (an internal standard—IS) in a concentration of 100 µg/mL. The use of the WS solution and DBGE with keeping the final volume of calibration solutions constant allowed a stable IS concentration at 100 µg/mL. Calibration solutions (6.25, 12.5, 25.0, 37.5, 50.0, 75.0 µg/mL; non-diluted WS was also used for calibration purposes) were prepared according to two methodologies: automatic (see Section 2.1. Development of the AHMT Procedure) and manual pipetting.

### 3.3. Calculation Parameters of the AHMT Procedure

The construction parameters (calculation of time and pressure values) of calibration solutions relied on maintaining constant total volume (sum of volumes of WS and DBGE) for all calibration solutions, with the assumption of equal viscosities of WS and DBGE. The study started by setting up the parameters for preparation of a 2-fold diluted WS for which time and pressure values for transfer WS and DBGE are the same. Then, construction of remaining dilutions, in practice, meant proportionally changing these parameters, ensuring that the sum of multiplications of time and pressure for WS and DBGE was constant for all prepared solutions (t_WS_·p_WS_ + t_DBGE_·p_DBGE_ = const.).

### 3.4. Instrumentation

Experiments were performed on the PA800 Plus Pharmaceutical Analysis System (Beckman Coulter, Fullerton, CA, USA) and P/ACE MDQ Capillary Electrophoresis System (Beckman Coulter, Fullerton, CA, USA), both equipped with a diode array detector (DAD). Signal acquisition was carried out at 210 nm. The uncoated bare fused-silica capillary was used. The capillary (ID 50 µm) was of 30.5 cm total length, 20.5 cm effective length. Background electrolyte (BGE) contained 103.9 mmol/L of H_3_PO_4_ and 37.66 mmol/L of TRIS. It was prepared in ultrapure water, filtered (0.45 µm regenerated cellulose membrane), and before the runs, degassed by centrifugation. Before the first separation run in each working day, the capillary was rinsed (137.90 kPa, 20 psi) with the sequence: 0.1 M NaOH (10 min), water (10 min), and BGE (2 min). For the fresh capillary, conditioning was: 1 M NaOH (20 min), water (3 min), methanol (10 min), and finally water (3 min). Between runs, the capillary was rinsed with water, methanol, and 0.1 M NaOH for 1.5 min and BGE for 2 min. Sample injection was conducted using forward pressure of 3.45 kPa (0.5 psi) for 6 s. During separations, the voltage of 15 kV (cathode at outlet) was applied without external pressure. The temperature of cooling liquid was set at 20ºC. Each measurement was performed in triplicate.

### 3.5. Data Treatment 

Electropherograms were collected and processed (e.g., Peak integration) in 32 Karat (v.9.1 build 10) Software (April, 2009; Beckman Coulter, Fullerton, CA, USA). Numerical data were processed in MS Excel (Office 365). Origin 2019b software (OriginLab Corporation, Northampton, MA, USA) was used for electropherogram plotting and statistical evaluation of obtained curves.

## 4. Conclusions

In this study, an automated hydrodynamically mediated technique (AHMT) for preparation of calibration solutions was developed and evaluated. Evaluation of calibration curves was performed in comparison to ones from manually prepared calibration solutions. The determination coefficients (r^2^) for all curves were higher than 0.997, and the employed evaluation protocol did not show significant differences between ones from this new approach and a manual calibration solution. The presented procedure in combination with the use of nanoVials enabled greater minimization of standard solution and solvent consumption (more than two times lower), while lowering the risk of errors related to pipetting tiny volumes of solution. This should not be neglected, especially in bioanalysis requiring use of expensive chemicals, such as proteins, etc. AHMT is a procedure that can be ran unsupervised overnight, thus limiting manpower consumption. One should notice that benefits from automatic operations are balanced by increased time consumption. AHMT requires about 2.5 h for preparation of six calibrators, which is related to the minimal time of solution transfer via a narrow bore capillary (10 min using pressure higher than 0.5 psi/3.45 kPa). Nevertheless, it should be emphasized that such automation does not entail any investment in new hardware or software, as the whole procedure can be achieved using commercially available CE systems. Additional profits from the use of AHMT, as well as possible limitations, will be a subject of our future work.

## Figures and Tables

**Table 1:** Parameters for preparation of calibration solutions by CE instrument using PCR vials and SCIEX^®^ nanoVials.

c (μg/mL)	PCR Vials				NanoVials			
	WS		DBGE		WS		DBGE	
	t (min)	p (psi/kPa)	t (min)	p (psi/kPa)	t (min)	p (psi/kPa)	t (min)	p (psi/kPa)
6.25	10	2.5 (17.24)	15	25 (172.37)	5	0.5 (3.45)	10	3.8 (26.20)
12.5	10	5.0 (34.47)	14	25 (172.37)	10	0.5 (3.45)	10	3.5 (24.13)
25.0	10	10 (68.95)	10	30 (206.84)	10	1.0 (6.90)	10	3.0 (20.68)
37.5	10	15 (103.42)	10	25 (172.37)	10	1.5 (10.34)	10	2.5 (17.24)
50.0	10	20 (137.90)	10	20 (137.90)	10	2.0 (13.79)	10	2.0 (13.79)
75.0	10	30 (206.84)	10	10 (68.95)	10	3.0 (20.68)	10	1.0 (6.90)

t—transfer time; p—transfer pressure.

**Table 2:** Calibration parameters obtained for prilocaine and bupivacaine using PCR vials and SCIEX^®^ nanoVials.

CE Device	Mode	Prilocaine			Bupivacaine		
		a	b	r^2^	a	b	r^2^
		PCR Vials					
PA800 Plus	R	0.0234	−0.0294	0.9988	0.0122	−0.0160	0.9987
	F	0.0230	−0.0198	0.9980	0.0119	−0.0133	0.9976
	M	0.0232	−0.0162	0.9990	0.0121	−0.0109	0.9987
P/ACE MDQ	R	0.0229	−0.0163	0.9991	0.0119	−0.0094	0.9989
	F	0.0229	−0.0088	0.9989	0.0119	−0.0077	0.9989
	M	0.0232	−0.0180	0.9994	0.0120	−0.0101	0.9993
	**Nano Vials**						
PA800 Plus	nV	0.0321	−0.0070	1.0000	0.0186	−0.0072	0.9999
	R	0.0312	−0.0193	0.9996	0.0181	−0.0118	0.9998
	M	0.0314	−0.0068	0.9999	0.0182	−0.0059	0.9998

R—‘PCR reverse’, F—‘PCR forward’, M—manually, nV—‘nanoVials reverse’, a—slope (mL/μg), b—intercept, r^2^—coefficient of determination.

**Table 3:** Deming regression parameters calculated for comparing the AHMT in forward (F) and reverse (R) modes and manual (M) approach with the use of PCR vials and SCIEX^®^ nanoVials in reversed mode (nV).

Ce Device	Mode	Prilocaine				Bupivacaine			
		a	S_a_ (10^−16^)	b	S_b_ (10^−14^)	a	S_a_ (10^−16^)	b	S_b_ (10^−14^)
		PCR Vials							
PA800 Plus	F vs. M	1.01	4.26	0.16	2.31	1.01	0.20	0.20	0.11
	R vs. M	0.99	0.19	0.56	0.10	0.99	6.46	0.42	3.51
P/ACE MDQ	F vs. M	1.01	6.45	−0.40	3.50	1.01	6.64	−0.20	3.60
	R vs. M	1.01	5.16	−0.08	2.79	1.01	7.81	−0.06	4.24
		**Nano Vials**							
PA800 Plus	nV vs. M	0.98	8.67	0.01	4.70	0.98	7.88	0.07	4.26
	nV vs. R	0.97	0.15	–0.38	8.08	0.97	0.12	–0.25	6.53

S_a_—standard error of the slope (mL/μg); S_b_—standard error of the intercept.

## Supplementary Materials

The following are available online, Figure S1: Photograph of a PCR vial placed in a buffer vessel and a SCIEX^®^ nanoVial, Table S1: Calculated concentration, c_k_ (μg/mL), theoretical concentration of the calibration solution, c_0_ (μg/mL), and relative error (RE%) values for reverse, forward, and manual modes, Table S2: Calculated concentration, c_k_ (μg/mL), theoretical concentration of the calibration solution, c_0_ (μg/mL), and relative error (RE%) values for manual approach and reverse mode using PCR vials and nanoVials.

## Sample Availability

Samples of the compounds procaine, prilocaine, bupivacaine in methanolic mixture solution are available from the authors.
