# Supplementary material for: An Automated Hydrodynamically Mediated Technique for Preparation of Calibration Solutions via Capillary Electrophoresis System as a Promising Alternative to Manual Pipetting
Source: Molecules. 2021 Oct 16;26(20):6268. doi: 10.3390/molecules26206268 (PMC8540481; doi:10.3390/molecules26206268)
Supplement: Supplementary file 1 [file molecules-26-06268-s001.zip › molecules-1403262-supplementary.pdf]

Supplementary

Table S1. Calculated concentration, c_k (μg/mL), theoretical concentration of the calibration solution, c_0 (μg/mL), and relative error, RE (%), values for reverse, forward and manual modes

CE device	Mode	c_0	c_k	$c_k - c_0$	RE	c_k	$c_k - c_0$	RE
			**prilocaine**			**bupivacaine**		
PA800 Plus	forward	6.25	6.76	0.51	8.17	6.91	0.66	10.48
		12.5	12.72	0.22	1.73	12.76	0.26	2.06
		25.0	24.83	-0.17	-0.67	24.86	-0.14	-0.54
		37.5	36.93	-0.57	-1.52	36.53	-0.97	-2.58
		50.0	49.85	-0.15	-0.31	50.05	0.05	0.10
		75.0	75.21	0.21	0.29	76.16	1.16	1.54
		100.0	103.56	3.56	3.56	103.80	3.80	3.80
	reverse	6.25	7.05	0.80	12.83	7.01	0.76	12.17
		12.5	12.90	0.40	3.23	12.76	0.26	2.10
		25.0	24.81	-0.19	-0.77	24.66	-0.34	-1.36
		37.5	36.69	-0.81	-2.15	36.13	-1.37	-3.66
		50.0	49.38	-0.62	-1.23	49.41	-0.59	-1.18
		75.0	74.31	-0.69	-0.92	75.07	0.07	0.09
		100.0	102.16	2.16	2.16	102.23	2.23	2.23
	manual	6.25	6.55	0.30	4.78	6.63	0.38	6.08
		12.5	12.46	-0.04	-0.35	12.42	-0.08	-0.64
		25.0	24.47	-0.53	-2.11	24.40	-0.60	-2.42
		37.5	36.47	-1.03	-2.74	35.94	-1.56	-4.16
		50.0	49.28	-0.72	-1.44	49.31	-0.69	-1.38
		75.0	74.44	-0.56	-0.74	75.14	0.14	0.18
		100.0	102.56	2.56	2.56	102.48	2.48	2.48
P/ACE MDQ	forward	6.25	6.20	-0.05	-0.83	6.42	0.17	2.76
		12.5	12.15	-0.35	-2.80	12.33	-0.17	-1.39
		25.0	24.30	-0.70	-2.80	24.46	-0.54	-2.17
		37.5	36.69	-0.81	-2.16	36.85	-0.65	-1.72
		50.0	49.41	-0.59	-1.19	49.58	-0.42	-0.83
		75.0	75.54	0.54	0.72	75.71	0.71	0.95
		100.0	102.38	2.38	2.38	102.66	2.66	2.66
	reverse	6.25	6.52	0.27	4.32	6.57	0.32	5.10
		12.5	12.47	-0.03	-0.25	12.48	-0.02	-0.18
		25.0	24.62	-0.38	-1.54	24.62	-0.38	-1.53
		37.5	37.00	-0.50	-1.34	37.03	-0.47	-1.27
		50.0	49.71	-0.29	-0.58	49.77	-0.23	-0.47
		75.0	75.83	0.83	1.11	75.92	0.92	1.22
		100.0	102.66	2.66	2.66	102.88	2.88	2.88
	manual	6.25	6.53	0.28	4.50	6.56	0.31	4.89
		12.5	12.42	-0.08	-0.63	12.40	-0.10	-0.79
		25.0	24.45	-0.55	-2.21	24.41	-0.59	-2.36
		37.5	36.71	-0.79	-2.12	36.68	-0.82	-2.18
		50.0	49.29	-0.71	-1.42	49.29	-0.71	-1.43
		75.0	75.15	0.15	0.21	75.16	0.16	0.21
		100.0	101.72	1.72	1.72	101.83	1.83	1.83

Table S2. Calculated concentration, c_k (μg/mL), theoretical concentration of the calibration solution, c_0 (μg/mL), and relative error, RE (%), values for manual approach and reverse mode using PCR vials and nanoVials

CE device	Mode	c_0	c_k	$c_k - c_0$	RE	c_k	$c_k - c_0$	RE
			**prilocaine**			**bupivacaine**		
PA800 Plus	nanoVials	6.25	6.22	-0.03	-0.44	6.28	0.03	0.41
		12.5	12.29	-0.21	-1.65	12.40	-0.10	-0.82
		25.0	24.03	-0.97	-3.87	24.12	-0.88	-3.51
		37.5	36.39	-1.11	-2.97	36.35	-1.15	-3.07
		50.0	48.72	-1.28	-2.57	48.75	-1.25	-2.49
		75.0	73.90	-1.10	-1.47	74.10	-0.90	-1.20
		100.0	98.95	-1.05	-1.05	98.22	-1.78	-1.78
	PCR vials	6.25	6.80	0.55	8.75	6.72	0.47	7.52
		12.5	13.05	0.55	4.36	13.03	0.53	4.23
		25.0	25.13	0.13	0.51	25.11	0.11	0.44
		37.5	37.84	0.34	0.91	37.71	0.21	0.55
		50.0	50.53	0.53	1.06	50.49	0.49	0.98
		75.0	76.45	1.45	1.93	76.61	1.61	2.15
		100.0	102.23	2.23	2.23	101.46	1.46	1.46
	manual	6.25	6.35	0.10	1.62	6.34	0.09	1.48
		12.5	12.56	0.06	0.44	12.60	0.10	0.79
		25.0	24.55	-0.45	-1.79	24.58	-0.42	-1.68
		37.5	37.18	-0.32	-0.86	37.07	-0.43	-1.13
		50.0	49.78	-0.22	-0.44	49.75	-0.25	-0.49
		75.0	75.51	0.51	0.69	75.66	0.66	0.88
		100.0	101.12	1.12	1.12	100.30	0.30	0.30

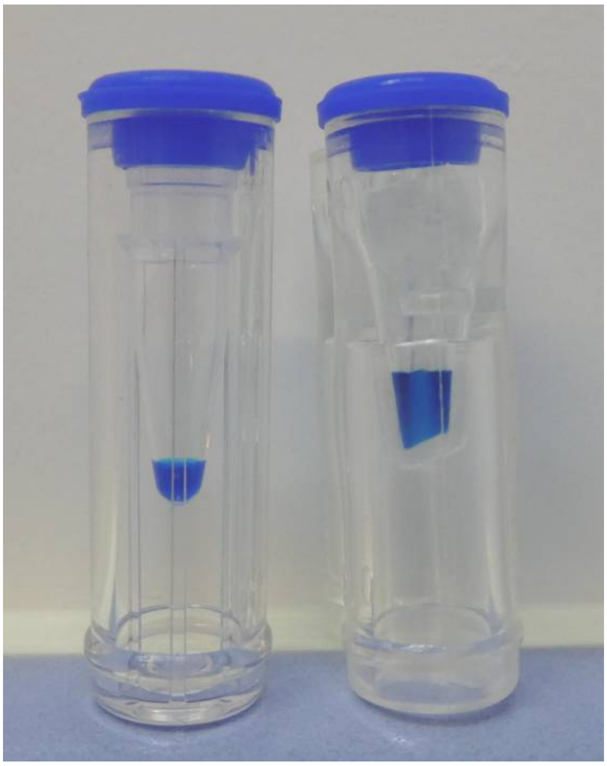

Figure S1. Photograph of a PCR vial placed in a buffer vessel (on the left) and a SCIEX® nanoVial (on the right). Both of them contain 10 μL of a liquid
